# Oncogenic MCT-1 activation promotes YY1-EGFR-MnSOD signaling and tumor progression

**DOI:** 10.1038/oncsis.2017.13

**Published:** 2017-04-10

**Authors:** H-Y Tseng, Y-A Chen, J Jen, P-C Shen, L-M Chen, T-D Lin, Y-C Wang, H-L Hsu

**Affiliations:** 1 Institute of Molecular and Genomic Medicine, National Health Research Institutes, Zhunan, Miaoli County, Taiwan; 2 Department of Pharmacology, National Cheng Kung University, Tainan, Taiwan

## Abstract

Tumor cells often produce high levels of reactive oxygen species (ROS) and display an increased ROS scavenging system. However, the molecular mechanism that balances antioxidative and oxidative stress in cancer cells is unclear. Here, we determined that oncogenic multiple copies in T-cell malignancy 1 (MCT-1) activity promotes the generation of intracellular ROS and mitochondrial superoxide. Overexpression of MCT-1 suppresses p53 accumulation but elevates the manganese-dependent superoxide dismutase (MnSOD) level via the YY1-EGFR signaling cascade, which protects cells against oxidative damage. Conversely, restricting ROS generation and/or targeting YY1 in lung cancer cells effectively inhibits the EGFR-MnSOD signaling pathway and cell invasiveness induced by MCT-1. Significantly, MCT-1 overexpression in lung cancer cells promotes tumor progression, necrosis and angiogenesis, and increases the number of tumor-promoting M2 macrophages and cancer-associated fibroblasts in the microenvironment. Clinical evidence further confirms that high expression of MCT-1 is associated with an increase in YY1, EGFR and MnSOD expression, accompanied by tumor recurrence, poor overall survival and EGFR mutation status in patients with lung cancers. Together, these data indicate that the MCT-1 oncogenic pathway is implicated in oxidative metabolism and lung carcinogenesis.

## Introduction

Reactive oxygen species (ROS) generated by cell metabolism can function either as signaling molecules or as cellular toxicants. As a double-edged sword, ROS influence signaling pathways to result in beneficial or detrimental outcomes in cancer therapy.^1, 2, 3^ Cellular ROS levels are balanced by scavenging systems such as superoxide dismutases (SODs), peroxiredoxins and glutathione peroxidas. Excessive ROS can damage proteins, lipids and DNA, leading to cell transformation and genetic mutations that contribute to carcinogenesis.

Cancer cells are capable of escaping oxidative stress by inducing antioxidant enzymes and molecules. ROS can trigger metabolic reprogramming of cancer cells to increase tumor aggressiveness and chemoresistance.^4, 5^ ROS released from cancer cells into the microenvironment can induce stromal oxidative stress and activate nuclear factor erythroid-2-related factor, hypoxia-inducible factor 1-alpha, vascular endothelial growth factor and nuclear factor kappa-light-chain-enhancer of activated B cells, which promote tumor immunity, inflammation and angiogenesis.^6, 7, 8^

p53 has a controversial role in ROS formation.^9^ ROS trigger p53 activation to regulate target genes and mediate p53-dependent apoptosis.^10^ Reciprocally, p53 controls cellular ROS generation by decreasing manganese superoxide dismutase (MnSOD) levels.^11, 12^ However, p53 also upregulates MnSOD and glutathione peroxidase, leading to an imbalance in antioxidant enzymes and an increase in oxidative stress.^13^ Aberrant induction of MnSOD sustains mitochondrial ROS generation and AMP-activated kinase signaling that promotes tumor progression toward an aggressive stage and causes therapeutic resistance and anti-apoptotic effects.^14, 15, 16^ Furthermore, overexpression of MnSOD promotes epithelial–mesenchymal transition in breast carcinoma,^17^ mediates tumor metastasis in nasopharyngeal carcinoma^18^ and elevates mitochondrial superoxide levels to activate PI3K/AKT and MMP2 in invasive pathways.^19, 20^

Multiple copies in T-cell malignancy 1 (MCT-1) is involved in post-transcriptional regulation and translation initiation.^21, 22, 23, 24^ We have previously shown that enhanced MCT-1 activity decreases the promoter function, protein stability and activity of p53; therefore, overexpression of MCT-1 advances tumorigenicity in a p53-null background.^25, 26, 27^ Multiple MCT-1 functions have been discovered that induce cell transformation and survival,^28, 29^ cause catastrophic mitosis^25, 30, 31^ and promote genomic instability.^25, 26^ In the MCT-1 oncogenic pathway, we have demonstrated that the expression of Shc (Src homology 2 domain-containing transforming protein) proteins is increased, which stimulates extracellular-regulated kinase and Ras signaling.^27, 29^ When epidermal growth factor receptor (EGFR) phosphorylates Shc, Shc forms a complex with Grb2-Sos to activate AKT, extracellular-regulated kinase, Ras and JNK pathways.^32, 33, 34^ In this study, we aimed to investigate whether the oncogenic activity of MCT-1 augments the EGFR signaling cascade and promotes ROS generation. We identified a novel carcinoma metabolism pathway involving the MCT-1-YY1-EGFR-MnSOD network, which confers oxidative resistance to oncogenic cells, accompanied by an enhancement in the tumor microenvironment and tumor progression.

## Results

### Overexpression of MCT-1 induces EGFR expression but inhibits p53 expression via YY1

**Figure 1:**
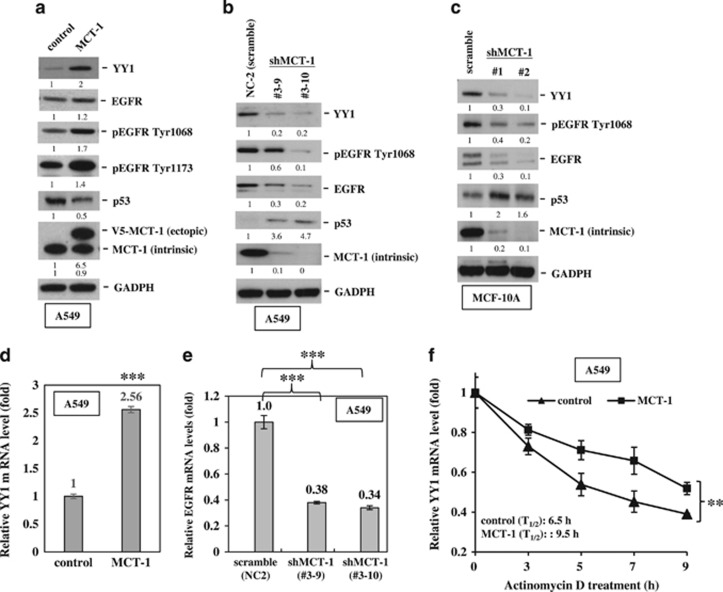
Overexpression of MCT-1 promotes YY1-EGFR signaling. MCF-10A cells and A549 cells overexpressing V5-tagged MCT-1 (V5-MCT-1) or with depleted endogenous MCT-1 (shMCT-1) and their comparative controls were analyzed. (**a**) The expression of YY1, EGFR and p53 was evaluated when A549 cells were starved for 24 h and then stimulated by EGF (20 ng/ml) and insulin (10 μg/ml) for 1 h. (**b**) Two different clones (#3–9 and #3–10) of MCT-1 knockdown and scrambled knockdown (NC2) in A549 cells were analyzed for YY1, EGFR and p53 expression upon EGF/insulin stimulation for 1 h. (**c**) MCF-10A cells with different degrees of MCT-1 knockdown (clone #1 and #2) and scrambled knockdown were examined after starvation for 24 h followed by EGF/insulin stimulation for 1 h. The protein amounts were normalized to GAPDH, and the phosphorylated EGFR levels were normalized to total EGFR before comparison with the comparative controls. (**d**) YY1 mRNA levels were surveyed in the A549 cells with different MCT-1 levels. (**e**) Relative EGFR mRNA levels were quantified in different A549 lines with MCT-1 knockdown (#3–9 and #3–10) and in A549 cells with scrambled knockdown vector (NC2). (**f**) The half-life (*T*_1/2_) of YY1 mRNA was examined in A549 cells after actinomycin D treatment. The data represent the mean±s.d. (*n*=3). **P<0.01 and ***P<0.001.

Yin Yang 1 (YY1) is a ubiquitous transcription factor overexpressed in many types of cancers and is associated with poor prognoses.^35, 36, 37^ YY1 regulates EGFR and p53 gene expression.^38, 39^ To study the oncogenic effect of MCT-1 in a wild-type p53 background, normal breast epithelial MCF-10A cells and invasive lung cancer A549 cells were transfected with a V5-tagged MCT-1 expression construct or the vehicle (control). We found that MCT-1 overexpression induced YY1 and EGFR but reduced p53 expression compared with control A549 cells (Figure 1a). Because of the increased amount of total EGFR, EGFR phosphorylation (Tyr1068 and Tyr1173) levels were also increased by MCT-1. However, the elevated levels of YY1 and EGFR were significantly reduced after targeting the MCT-1 gene (shMCT-1; clone #3–9 and #3–10) in A549 cells with a short hairpin RNA (shRNA) compared with cells containing scrambled shRNA (Figure 1b). Similarly, YY1 and EGFR were repressed but p53 was accumulated in the MCF-10A cells after MCT-1 depletion (shMCT-1, clone #1 and #2; Figure 1c). Consistent with the enrichment in YY1 protein induced by MCT-1 overexpression, the YY1 messenger RNA (mRNA) level also exhibited a 2.56-fold increase over the control cells (*P*<0.001; Figure 1d). Conversely, knockdown of MCT-1 in A549 cells (clones #3–9 and #3–10) repressed EGFR mRNA expression (Figure 1e). Actinomycin D was then used to inhibit transcription and to examine the steady-state of YY1 mRNA levels (Figure 1f). The results showed that YY1 mRNA had a longer half-life (*T*_1/2_=9.5 h) in the MCT-1-overexpressing cells than in control A549 cells (*T*_1/2_=6.5 h), indicating that MCT-1 induced YY1, at least in part, through stabilization of YY1 mRNA.

**Figure 2:**
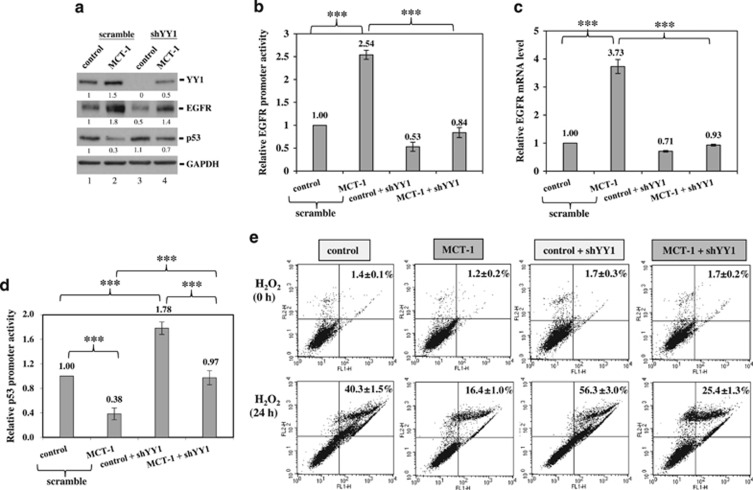
MCT-1 overexpression protects cells against oxidative stress via YY1. MCF-10A cells (control and MCT-1) with YY1 knockdown (shYY1) or scrambled knockdown were studied. (**a**) EGFR and p53 expression were analyzed in cells with different MCT-1 and YY1 levels. The protein amounts were normalized to GAPDH and compared with scrambled control (lane 1). (**b**) Relative EGFR promoter activity is indicated. (**c**) Relative EGFR mRNA level is shown. (**d**) Relative p53 promoter activity is shown. (**e**) After the cells were exposed to 100 μm H_2_O_2_ for 24 h, apoptotic events were evaluated using FITC-Annexin V staining and propidium iodide (PI) counter staining, followed by flow cytometry analysis. The data represent the mean±s.d. (*n*=3). ***P<0.001.

To inspect the role of YY1 in the regulation of EGFR and p53, the YY1 gene was knocked down using an shRNA (shYY1) to suppress YY1 expression, which resulted in EGFR reduction and p53 accumulation in the MCT-1-overexpressing cells (Figure 2a, lane 4). To further study whether YY1 mediates EGFR gene activation in the MCT-1 pathway, the EGFR promoter segment (–1102 to –12) was cloned into a pGL3 promoter-less vector. The data indicated that EGFR promoter activity in MCT-1-overexpressing cells was increased 2.54-fold over that in the control cells (*P*<0.001; Figure 2b), but it was repressed by YY1 knockdown (0.84-fold, *P*<0.001). Likewise, the EGFR mRNA level promoted by MCT-1 (3.73-fold, *P*<0.001) was inhibited after YY1 reduction (0.93-fold, *P*<0.001) compared with control cells (Figure 2c). Furthermore, the p53 promoter segment (−188 to +23) was cloned into a pGL3 basic vector, and the promoter activity was significantly inhibited upon MCT-1 induction (0.38-fold, *P*<0.001) but induced after YY1 knockdown (0.97-fold, *P*<0.001) compared with scrambled knockdown (Figure 2d). These results confirm that YY activates EGFR gene expression but inhibits p53 gene expression in the MCT-1 pathway.

To determine whether p53 regulates YY1 and EGFR in the MCT-1 pathway, the expression of p53 mRNA was abrogated with shRNA in MCF-10A cells (Supplementary Figure 1a). The EGFR promoter activity (Supplementary Figure 1b), the EGFR mRNA level (Supplementary Figure 1c) and the expression of EGFR and YY1 protein (Supplementary Figure 1d, lane 4) were additively enhanced as p53 was depleted from the MCT-1-overexpressing cells (MCT-1/-p53) compared with the p53-deficient control cells (control/-p53). These results indicate that YY1 and p53 antagonize each other in the MCT-1 pathway.

### MCT-1 cooperates with YY1 and EGFR to protect cells against oxidative stress

H_2_O_2_ exposure directly increases intracellular ROS levels.^40^ To determine whether YY1 is important for protecting cells against oxidative stress, MCF-10A cells were exposed to H_2_O_2_ for 24 h. The data showed that the MCT-1-overexpressing cells were more refractory to oxidative cell death (16.4±1.0%) than the control cells (40.3±1.5% Figure 2e), as detected by fluorescein isothiocyanate (FITC)-Annexin V staining and flow cytometry analysis to assess apoptosis levels. Clearly, YY1 functionally protected the cells against oxidative damage because the number of apoptotic events increased as YY1 was depleted from the control cells (56.3±3.0%) and the MCT-1-overexpressing cells (25.4±1.3%). Similarly, the MCT-1-induced A549 cell survival was also suppressed after targeting YY1 (shYY1), as evidenced by the increased apoptotic DNA fragmentation observed in the TUNEL assay (Supplementary Figure 2a).

**Figure 3:**
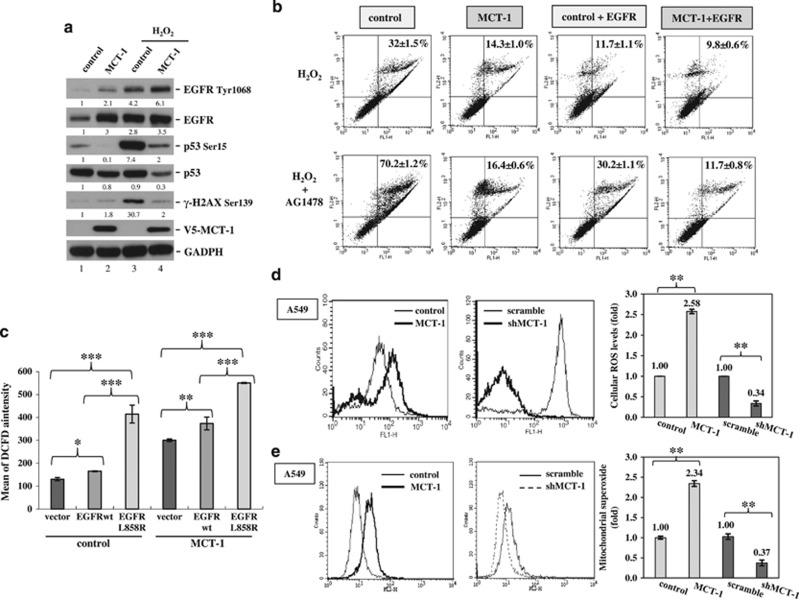
MCT-1 and EGFR co-operatively protect cells against oxidative stress. MCF-10A (**a**–**c**) and A549 cells (**d**, **e**) with different levels of MCT-1 expression were studied. (**a**) The expression and phosphorylation levels of EGFR, p53 and H2AX were studied after H_2_O_2_ exposure for 1 h. The protein amounts were normalized to GAPDH, and the phosphorylated EGFR levels were normalized to total EGFR before comparison with the comparative control (lane 1). (**b**) The cells (control, MCT-1) with or without EGFR co-expression were exposed to H_2_O_2_ for 24 h or pre-treated with 5 μm AG1478 for 1 h. Flow cytometry was used to analyze the oxidative cell death after FITC-Annexin V staining and propidium iodide (PI) counter staining. (**c**) ROS generation in the cells overexpressing wild-type EGFR (EGFRwt) or mutant EGFR (L858R) was detected by a DCFDA-cellular ROS method. (**d**) Intracellular ROS levels were analyzed and compared in different cellular contents (MCT-1 overexpressing vs vector control and MCT-1 silencing (shMCT-1) vs. scrambled knockdown). (**e**) Mitochondrial superoxide levels were examined and quantified in different MCT-1 contents using mitochondrial superoxide indicator (MitoSOX) and flow cytometry analysis. Data represent the mean±s.d. (*n*=3). *P<0.05; **P<0.01; ***P<0.001.

Upon further examination of the MCF-10A cellular response to oxidative stress (Figure 3a), the activating phosphorylation of EGFR (Tyr1068) was additively promoted by H_2_O_2_ exposure and MCT-1 overexpression, whereas the activating phosphorylation of p53 (Ser15) and the DNA damage marker γ-H2AX (Ser139) were reduced when MCT-1 was overexpressed (lane 4) compared with the control group (lane 3). A reduced DNA damage response was also observed in the MCT-1 oncogenic cells when exposed to H_2_O_2_ and when AG1478 inactivated EGFR phosphorylation (Tyr1068) (Supplementary Figure 2b), as indicated by the decrease in γ-H2AX. Therefore, MCT-1 overexpression protects cells against oxidative DNA damage.

To inspect whether EGFR and MCT-1 work together against oxidative stress, wild-type (wt) EGFR was introduced to promote the oncogenic effect in MCF-10A cells (Supplementary Figure 2c). As evaluated by FITC-Annexin V staining and observation of apoptotic events (Figure 3b), H_2_O_2_-induced apoptotic effects were less frequent in the MCT-1-expressing group (14.3±1%) than in the control cohort (32±1.5%). However, when EGFR was overexpressed (+EGFR), oxidative cell death was relatively decreased in MCT-1-overexpressing cells (9.8±0.6%) and significantly inhibited in control cells (11.7±1.1%). Conversely, when AG1478 inactivated EGFR (+AG1478), H_2_O_2_-induced apoptotic events were dramatically promoted in control cells (70.2±1.2%) but not in MCT-1-overexpressing cells (16.4±0.6%). Even after treatment with AG1478, the cells co-induced with MCT-1 and EGFR were still more refractory to H_2_O_2_ (MCT-1+EGFR, 11.7±0.8%) than the comparative control (control+EGFR, 30.2±1.1%). Hence, EGFR and MCT-1 co-operatively protect cells against oxidative injury.

### MCT-1 promotes intracellular ROS generation

EGFR-activating mutations and oxidative stress morphologically transform MCF-10A cells and enhance their oncogenic properties.^37, 41^ EGFR overexpression and an EGFR-activating mutation (L858R) have been observed in triple-negative breast cancer (TNBC).^42^ EGFRwt and the L858R mutant were introduced into MCF-10A cells to study whether EGFRwt or the L858R mutant affect ROS generation in normal breast epithelial cells (Figure 3c). Using a DCFDA-cellular ROS detection system, we found that the intracellular ROS levels were enhanced when EGFRwt was co-induced with MCT-1, and ROS levels were further advanced when MCT-1 oncogenic cells expressed the EGFR L858R mutant. This result suggests that MCT-1 overexpression alongside EGFR overexpression or expression of mutated EGFR may develop a ROS scavenging system to adapt to an oxidative environment and maintain oncogenic cell survival.

Cancer cell metabolism continually produces superoxide (O_2_^−^) and hydrogen peroxide (H_2_O_2_).^43^ Upon further examination of the intrinsic ROS production in A549 cancer cells, we noted a 2.58-fold increase in the intracellular ROS level in MCT-1-overexpressing cells over the control group (*P*<0.01; Figure 3d). In contrast, ROS levels exhibited a 2.94-fold reduction after MCT-1 knockdown (shMCT-1) compared with scrambled knockdown (*P*<0.01). Mitochondria are major sources of ROS generation in cancer cells.^1^ Using mitochondrial superoxide indicator (MitoSOX) Red to detect the superoxide level in mitochondria, a 2.34-fold elevation in mitochondrial superoxide levels was identified in MCT-1-overexpressing cells (Figure 3e), whereas mitochondrial ROS showed a 2.7-fold reduction after MCT-1 knockdown. Therefore, MCT-1 oncogenic stress stimulates the formation of intracellular and mitochondrial ROS. Loss of MCT-1 effectively alleviates ROS production, presumably by improving an antioxidant mechanism.

### MCT-1 induces MnSOD via YY1-EGFR signaling

**Figure 4:**
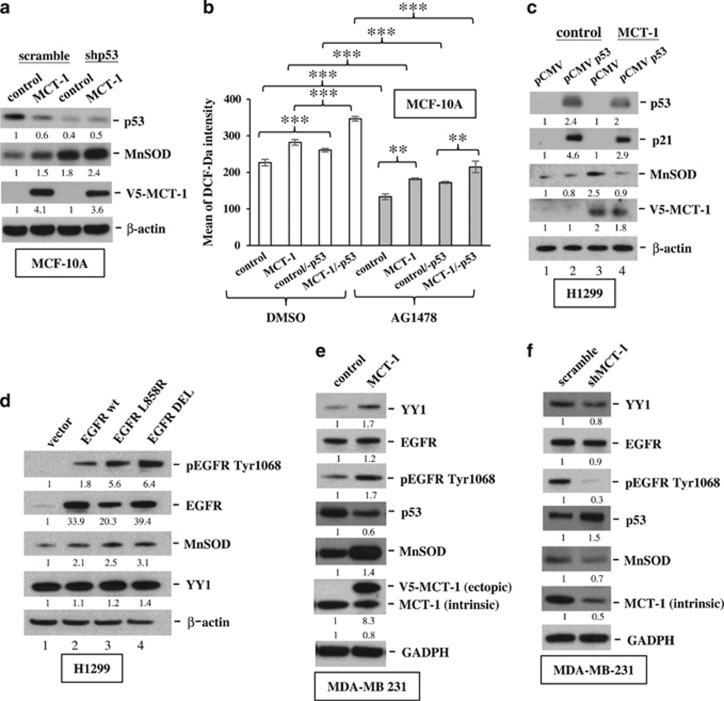
MnSOD is induced by MCT-1 overexpression, p53 knockdown and EGFR activation. (**a**) MnSOD expression was examined in MCF-10A cells (control and MCT-1) with (shp53) or without (scramble) p53 depletion. (**b**) Cellular ROS levels were analyzed in MCF-10A cells with different conditions of p53 and MCT-1 expression upon DMSO or AG1478 treatment. Data represent the mean±s.d. (*n*=3). **P<0.01; ***P<0.001. (**c**) MnSOD levels were characterized in the H1299 cells (control and MCT-1) without (pCMV) or with p53 re-expression (pCMV p53). (**d**) The EGFR phosphorylation and MnSOD levels were assessed when H1299 cells were introducing empty vector, wild-type EGFR and EGFR-activating mutants (L858R and exon19 DEL). (**e**) MDA-MB231 cells with or without MCT-1 overexpression were assayed. (**f**) MDA-MB231 cells with or without MCT-1 knockdown were evaluated. The protein amounts were normalized to β-actin or GAPDH, and the phosphorylated EGFR levels were normalized to total EGFR levels before comparison with the comparative controls (lane 1).

Mitochondrial superoxide generation stimulates MnSOD expression in cancer cells.^44^ We investigated MnSOD expression in MCF-10A cells and found that overexpressing MCT-1 increased MnSOD but decreased p53 levels (Figure 4a), but knockdown of p53 (shp53) further increased the amount of MnSOD in MCT-1 oncogenic cells. Correspondingly, cellular ROS levels elevated by MCT-1 were enhanced via p53 knockdown (Figure 4b), but they were significantly suppressed by EGFR inactivation (AG1478). When H1299 cells (p53-null) were induced to re-express p53 (pCMV p53; Figure 4c), the MCT-1-induced MnSOD level was significantly repressed (lane 4). Moreover, the extent of the increase in MnSOD corresponded to the advanced EGFR activity of the EGFR-activating mutants (L858R and exon19 deletion (DEL); lanes 3 and 4) compared with EGFRwt overexpression (lane 2) in H1299 cells (Figure 4d). Intriguingly, MnSOD was also induced in TNBC MDA-MB231 cells when MCT-1 overexpression induced YY1 induction, EGFR phosphorylation (Tyr1068) and decreased p53 levels (Figure 4e). Conversely, MnSOD was decreased, accompanied by EGFR de-phosphorylation (Tyr1068) and p53 accumulation when MCT-1 was targeted in MDA-MB231 cells (Figure 4f). Accordingly, MnSOD expression is determined by MCT-1 expression, p53 function and EGFR activation.

**Figure 5:**
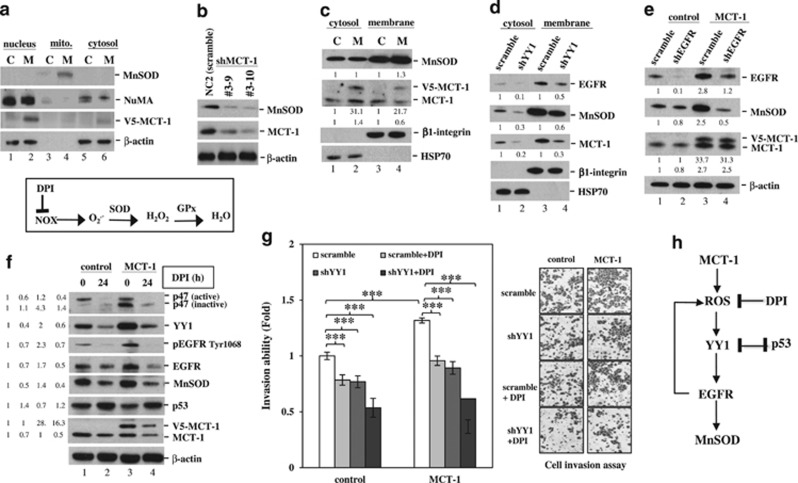
MCT-1 induces MnSOD expression and promotes cancer cell invasion via the YY1 pathway. The A549 cancer cells were studied. (**a**) Subcellular distributions of MnSOD, NuMA, MCT-1 and β-actin in the nucleus, mitochondria (mito.) and cytoplasm were characterized in control (C) and MCT-1-overexpressing (M) cells. (**b**) MnSOD expression levels were examined in different MCT-1-silencing clones (#3–9 and #3–10) and scrambled knockdown (NC2). (**c**) The distribution of MnSOD and MCT-1 in the cytosolic and membrane fractions was studied. HSP70 and β1-integrin were the fraction markers. The amounts of cytosolic and membrane proteins were normalized to HSP70 and β1-integrin, respectively, and then compared with scrambled knockdown (lanes 1 and 3). (**d**) The effects of YY1 knockdown (shYY1) on the distribution of EGFR, MnSOD, MCT-1 and HSP70 in the cytosol and membrane were studied. The cytosol and membrane protein amounts were normalized to HSP70 and β1-integrin, respectively, and then compared with scrambled knockdown (lanes 1 and 3). (**e**) The effects of EGFR knockdown (shEGFR) on MnSOD and MCT-1 expression were assessed. The protein amounts were normalized to β-actin before comparison with scrambled control (lane 1). (**f**) The influence of DPI (an ROS inhibitor) on the expression of p47^phox^ (active and inactive forms), YY1, EGFR, MnSOD and p53 was studied. The protein amounts were normalized to β-actin before comparison with the comparative control (lane 1). (**g**) Cell invasiveness affected by DPI treatment and YY1 knockdown was evaluated in different MCT-1 expression conditions. The data represent the mean±s.d. (*n*=3). ***P<0.001. (**h**) A proposed model of how MCT-1 overexpression promotes ROS generation and stimulates YY1-EGFR-MnSOD signaling, which can be suppressed by DPI and p53.

When MnSOD distribution was analyzed in the nuclear, mitochondrial (mito.) and cytosol fractions, we observed that the majority of MnSOD was located in the mitochondria, and a higher MnSOD level was found in MCT-1-overexpressing cells (M) than in control A549 cells (C; Figure 5a, lane 4). Conversely, MnSOD levels were diminished equivalently to the extent of MCT-1 knockdown (clone #3–9 and #3–10; Figure 5b). MnSOD is a membrane-bound protein.^45^ Further evaluation of the MnSOD location in the cytoplasmic and membrane (mitochondria-containing) fractions revealed that only membrane-bound MnSOD was significantly elevated upon MCT-1 overexpression (Figure 5c), which may affect the redox metabolism of the oncogenic cells.

Although YY1 regulates mitochondrial complex I genes,^46^ and YY1 deficiency disrupts the structure and function of mitochondria, it is unknown whether YY1 affects MnSOD expression in cancer cells. In the subcellular fractionation analysis, YY knockdown (shYY1) in A549 cells showed a substantial inhibitory effect on EGFR and MnSOD in both the cytosolic and membrane compartments (Figure 5d, lanes 2 and 4) compared with scrambled knockdown. The MCT-1 promoter contains 10 putative YY1-binding sites (Supplementary Figure 2d). Consistent with this finding, loss of YY1 reduced MCT-1, indicating that positive regulatory feedback exists between YY1 and MCT-1. Similar to the suppression of cellular MnSOD by silencing of YY1 (+shYY1; Supplementary Figure 2e), targeting EGFR (+shEGFR) also markedly inhibited the increase in MnSOD in MCT-1 oncogenic cells (Figure 5e, lane 4). These data demonstrate, for the first time, that the MCT-1-YY1-EGFR pathway modulates MnSOD expression in cancer cells.

The phosphorylation of p47^phox^ enhances NADPH oxidase (NOX) activation and superoxide production.^47^ Diphenyleneiodonium (DPI), a ROS inhibitor, abolishes NOX-mediated superoxide (O_2_^−^) formation, which suppresses intracellular ROS levels.^3^ Intriguingly, we found that MCT-1 stimulated the expression of p47^phox^, accompanied by enrichment of YY1, EGFR and MnSOD, in A549 cells (Figure 5f). However, DPI treatment not only reduced MCT-1 but also decreased p47^phox^, YY1, EGFR and MnSOD (lane 4). These results suggest that MCT-1 enhances ROS generation and mitochondria superoxide formation, possibly by deregulating activities of oxidase (NOX) and antioxidant (MnSOD).

To explore the influence of YY1 and ROS on cancer cell invasiveness, we found that shYY1 and DPI suppressed the A549 cell invasiveness induced by MCT-1 (Figure 5g); thus, the combinatory effect (shYY1+DPI) strongly prohibited cancer cell invasion. The results from these studies reveal that MCT-1 overexpression induces ROS generation (Figure 5h), accompanied by YY1-EGFR-MnSOD signaling amplification and cancer invasiveness, which can be suppressed by DPI and p53 activity.

### Overexpression of MCT-1 promotes tumor progression and transforms the tumor microenvironment

**Figure 6:**
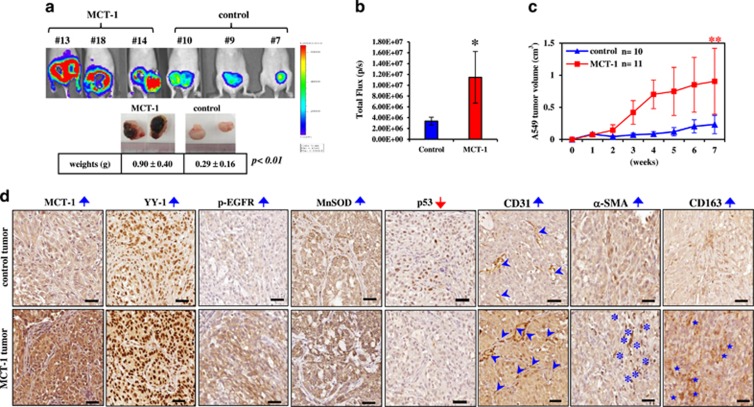
MCT-1 overexpression enhances A549 tumor progression. (**a**) Bioluminescent A549 cells were subcutaneously injected into nude mice, and tumors were allowed to develop for 8 weeks. Tumor expansion was detected by IVIS, and tumor weights were measured. (**b**) Photon flux detected by IVIS indicated tumorigenic outcomes at week 8. (**c**) Tumor growth was assessed weekly, and tumors in the xenograft mice injected with the control cells (*n*=10) or MCT-1-overexpressing cells (*n*=11) were compared. The data represent the mean±s.d. *P<0.05; **P<0.01. (**d**) Immunohistochemistry results indicated the amounts of MCT-1, YY1, p-EGFR, MnSOD and p53 in the A549 tumors. Tumor angiogenesis (CD31, denoted by arrowheads) as well as the accumulation of myofibroblasts (α-SMA, indicated by asters) and tumor-promoting M2 macrophages (CD163, indicated by stars) were also characterized. Scale bar represents 50 μm.

To determine whether the MCT-1 oncogenic pathway can alter the microenvironment(s) to benefit tumor progression, bioluminescent A549 cells were subcutaneously injected into BALB/c nude mice, and tumors were allowed to develop for 8 weeks. Extensive tumor necrosis and increased tumor burdens were observed in mice-bearing MCT-1-overexpressing cells (Figure 6a). An *in vivo* bioluminescence imaging system (IVIS) also detected a higher photon flux (Figure 6b), indicative of the advanced tumor growth underlying MCT-1 oncogenicity (Figure 6c).

A tumor necrotic cascade initiated by the oncogenic effect and oxidative stress would distort the function and structure of mitochondria.^48, 49^ MCT-1 oncogenic activity promotes tumor necrosis, which may elicit an inflammatory response and a malignant microenvironment for tumor progression.^50, 51^ To inspect changes in the tumor microenvironment, the angiogenesis marker CD31 (denoted by arrowheads), the cancer-associated fibroblast marker α-SMA (marked by asterisks) and the tumor-associated M2 macrophage marker CD163 (indicated by stars) were examined in an immunohistochemistry study (Figure 6d). Surprisingly, we observed that tumor angiogenesis (CD31), cancer-associated fibroblasts (α-SMA) and M2 macrophages (CD163) were all enriched, with increased YY1, p-EGFR (Tyr1068) and MnSOD expression but low p53 expression, in the MCT-1-overexpressing tumors compared with control tumors. These data reveal that MCT-1 promotes tumor progression, necrosis and the tumor-promoting activity of the microenvironment alongside the enhancement of YY, EGFR and MnSOD *in vivo*.

### Clinical relevance of MCT-1, YY1, EGFR, MnSOD and p53 in human lung cancer

**Figure 7:**
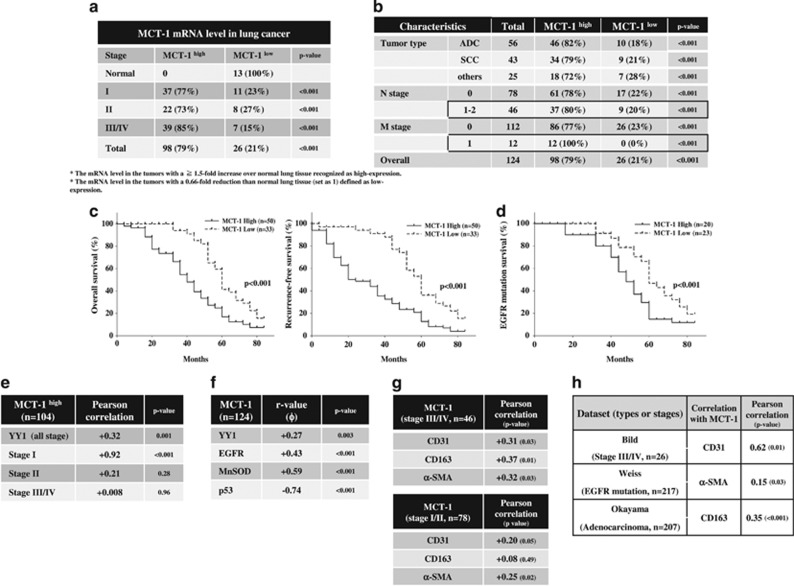
Clinical relevance of MCT-1 and its relationship with YY1, EGFR, MnSOD, CD31, CD163 and α-SMA in human lung cancer. (**a**) MCT-1 mRNA levels in lung cancer tissue (*n*=124) and normal lung tissue (*n*=13) were studied with the TissueScan arrays. The MCT-1 mRNA level in each tumor sample was normalized to the β-actin mRNA level and then calibrated to the average MCT-1 mRNA level in normal lung tissue. *X*^2^ tests were used to evaluate the significance of MCT-1 overexpression at different tumor stages compared with the normal tissue. (**b**) MCT-1 mRNA levels were assessed in different tumor types, in lymph node metastasis (*N*⩾1) and in distant metastasis (*M*=1) using *X*^2^ tests. (**c**) The correlation between MCT-1 expression levels and overall survival and recurrence-free survival (*n*=83) were analyzed using the Okayama dataset in the ONCOMINE database. (**d**) The overall survival of patients with an EGFR mutation associated with MCT-1 expression levels was analyzed in the Okayama dataset (*n*=43). (**e**) Pearson coefficient correlation defines the linkage between MCT-1 overexpression and YY1 (*n*=104). (**f**) The Phi coefficient shows the relationship of MCT-1 with YY1, EGFR, MnSOD and p53 in lung cancer patients (*n*=124). (**g**) Pearson coefficient correlation reveals the association between MCT-1 with CD31, CD163 and α-SMA in the lung cancers at stage III/IV (*n*=46) and at stage I/II (*n*=78). (**h**) Pearson coefficient correlation indicates the association between MCT-1 and CD31, CD163 and α-SMA in different types and stages of lung cancer in the ONCOMINE database (Bild, *n*=26; Weiss, *n*=217; Okayama, *n*=207).

Lung cancer TissueScan qPCR arrays (OriGene Technologies, Inc.) were used to investigate the clinical relevance of MCT-1, YY1, EGFR, MnSOD and p53 expression in lung cancer patients. The results showed that MCT-1 mRNA levels were often induced in lung cancers (*n*=124) at stage I (77%, *P*<0.001), stage II (73%, *P*<0.001) and stage III-IV (85%, *P*<0.001; Figure 7a), which displayed a 1.5-fold increase over the mean MCT-1 mRNA level in normal lung tissue (*n*=13). Overall, MCT-1 was highly expressed in 79% of the patients with adenocarcinoma (82% Figure 7b), squamous cell carcinoma (79%) and other cell type (small- and large-cell; 72%) lung cancers. High MCT-1 levels were also identified in patients with lymph node metastasis (*N*⩾1, *n*=46; 80%, *P*<0.001) and distal metastasis (*M*=1, *n*=12; 100%, *P*<0.001) compared with those without lymph node metastasis (78%, *P*<0.001) and distal metastasis (77%, *P*<0.001).

Using the ONCOMINE (www.oncomine.org) lung cancer database and data-mining platform (Supplementary Figure 3), high MCT-1 mRNA levels were also detected in different types of lung cancers in Hou’s data (*n*=156) as well as in Okayama’s lung adenocarcinoma data (*n*=246, *P*=2.29E-19) and Wei’s dataset (*n*=50, *P*=1.10E-11). Further surveying the Okayama dataset in an 84 month follow-up study (*n*=83; Figure 7c),^52^ high MCT-1 levels were found to be significantly correlated with poor overall survival (*P*<0.001) and low recurrence-free survival (*P*<0.001) compared with the patients with low MCT-1 levels. In an analysis of the Okayama data (Figure 7d), high MCT-1 levels were linked with poor survival of the patients with EGFR mutations (exon19 (DEL) and L858R) more than low MCT-1 levels (*P*<0.001). Accordingly, MCT-1 overexpression may be recognized as a biomarker for early detection and as a prognostic index in human lung carcinogenesis.

In contrast, only 50% of the cancer patients displayed high YY1 levels, and they were more prevalently identified in stage I (67%, *P*<0.001) cancer than in stage II (37%, *P*<0.01) and stage III/IV (41%, *P*<0.01) cancer relative to normal lung tissues (Supplementary Figure 4a). In addition, 70% of the patients expressed high EGFR levels (*P*<0.001; Supplementary Figure 4b), and 71% of the patients exhibited MnSOD induction (*P*<0.001; Supplementary Figure 4c), whereas the majority of cancer patients (92%) had p53 downregulation (*P*<0.001) (Supplementary Figure 4d). The log_2_ ratio of MCT-1, p53, EGFR and MnSOD mRNA levels compared with β-actin mRNA levels further validated the significant differences between lung tumors (T) and normal lung tissues (N; Supplementary Figure 4e).

To characterize the clinical relationship between MCT-1 and YY1, EGFR, MnSOD and p53, the average mRNA level of each gene in normal lung tissue was defined as the threshold. For a given gene, its expression level was dichotomized into ‘low’ and ‘high’ it was ‘high’ if and only if it was higher than the normal tissue. Consequently, both MCT-1 and YY1 were found to often be induced in patients at stage I (Pearson correlation +0.92, *P*<0.001; Figure 7e), suggesting their strong association with the initial step of lung tumorigenesis. MCT-1 expression was largely positively associated with YY1 (Phi coefficient +0.27, *P*=0.003), EGFR (Phi coefficient +0.43, *P*<0.001) and MnSOD (Phi coefficient +0.59, *P*<0.001) in all stages of lung cancers (Figure 7f). However, MCT-1 had a strong negative correlation with p53 expression (Phi coefficient −0.74, *P*<0.001). As a result, enhanced MCT-1 activation is associated with increased YY1, EGFR and MnSOD but decreased p53 in lung carcinogenesis.

Furthermore, the association between MCT-1 with CD31, CD163 and α-SMA gene expression was characterized using Lung cancer TissueScan qPCR arrays (Figure 7g), and the results showed that MCT-1 was positively correlated with CD31 (Pearson coefficient +0.31, *P*=0.03), CD163 (Pearson coefficient +0.37, *P*=0.01) and α-SMA (Pearson coefficient +0.32, *P*=0.03) in stage III/IV lung cancers. However, MCT-1 elevation was less associated with CD31 (Pearson coefficient +0.2, *P*=0.05) and α-SMA (Pearson coefficient +0.25, *P*=0.02) and was unrelated to CD163 (Pearson coefficient +0.08, *P*=0.49) in stage I/II patients.

Based on an analysis of the ONCOMINE database (Figure 7h), high MCT-1 levels were positively correlated to CD31 (Pearson correlation +0.62, *P*=0.01), α-SMA (Pearson correlation +0.15, *P*=0.03) and CD163 (Pearson correlation +0.35, *P*<0.001) expression in stage III/IV and different types of lung cancers. Collectively, MCT-1 overexpression is implicated in lung carcinogenesis and development of the tumor microenvironment.

## Discussion

Targeted therapies have achieved notable successes in some cancers. However, many targeted therapies are highly toxic to normal cells, and most patients experience relapse due to genetic mutation and tumor heterogeneity, whereby the tumors contain therapy-resistant stem cells that have adopted alternative and compensatory pathways. A combination of a ROS inhibitor with the suppression of an oncogenic pathway may be a low-toxic ‘broad-spectrum’ therapeutic approach that simultaneously targets many key pathways and mechanisms to alter the tumor microenvironment and prevent metastasis.^53^

We now demonstrate that oncogenic MCT-1 activation elevates ROS generation and amplifies YY1-EGFR-MnSOD signaling, accompanied by tumor promotion and a malignant microenvironment. Clinical data confirm the important connection between MCT-1 activity and YY1, EGFR and MnSOD, as well as the development of malignant microenvironments in lung carcinoma. Targeting the MCT-1 pathway may alter oxidative metabolism and reduce tumor aggressiveness.

Elevated ROS-mediated regulation of the T-cell immune response in the microenvironment is related to tumor-induced immunosuppression.^54^ Thus, T-cell-based cancer therapy combined with a ROS scavenger has been considered a promising strategy to improve T-cell immunity. ROS released from cancer cells potentially promote stromal fibroblast transformation into myofibroblasts that support tumor progression and dissemination.^55^ Thus, using antioxidants as an adjuvant therapy to modify tumor immunity and the microenvironment may lead to beneficial clinical outcomes.

MCT-1 enhances EGFR expression, possibly via a cooperative increase in YY1 (Figure 1) and SP1 as previously described.^28, 56, 57^ MicroRNA-34a (miR-34a) is induced by p53 and directly targets YY1 to inhibit YY1-induced and EGFR-mediated carcinogenesis and metastasis.^58, 59, 60, 61^ Loss of p53 amplifies MCT-1-YY1-EGFR signaling (Supplementary Figure 1) and induces MnSOD (Figure 4a), which may produce excessive ROS that enhance genetic mutation and tumor progression. Furthermore, MCT-1 may potentiate tumor angiogenesis by antagonizing the p53-miR-34a axis and inhibiting the angiogenesis inhibitor thrombospondin-1 (TSP1).^24, 62^

Under oncogenic stress, mitochondrial ROS overproduction can accelerate mutagenesis, which amplifies tumorigenic signaling and increases metastatic potential.^63^ Overexpression or activating-mutation of EGFR promotes tumor progression and metastasis.^64^ EGFR activity is implicated in DNA repair.^65^ Therefore, advanced ROS generation and increased cell survival via the combined effect of MCT-1 and EGFR may mis-repair oxidative DNA damage and promote growth of gene-mutated cells.

EGFR overexpression and mutation are frequently identified in metastatic non-small-cell lung cancer and are associated with poor prognosis.^66^ L858R is the most prevalent EGFR tyrosine kinase activating mutation. Mutation of the kinase domain in EGFR leads to a ligand-independent tyrosine kinase activation. EGFR activation is crucial for ROS production because deactivation of EGFR by AG1478 suppresses the generation of ROS (Figure 4b), explaining why ROS levels are higher in L858R mutant than wild-type EGFR cells (Figure 3c). The cells expressing wild-type EGFR have a higher ability to repair cisplatin- and IR-induced DNA damage than the L858R-mutant-expressing cells;^67^ therefore, the intracellular ROS levels are reduced because wild-type EGFR repairs the oxidative DNA damage.

Compared with other subtypes of breast cancer, EGFR expression, gene amplification and mutations are more frequently identified and associated with poor prognosis in TNBCs.^42, 68, 69^ For example, activating mutations in the EGFR gene (exon19 deletion and L858R and T790M mutations) are present in the tumors of TNBC patients.^68, 70^ Therefore, EGFR may also be a therapeutic target in TNBCs.

Enhanced MCT-1 activity induces the superoxide scavenger MnSOD and ROS generators, including NADPH (p47^phox^; Figure 5) and Shc (p66),^29^ explaining why the MCT-1 oncogenic cells survive and are capable of adapting to the oxidative environment (Figures 2e and 3b). Here, we show that MnSOD expression is stimulated by MCT-1 overexpression, p53 deficiency and EGFR activation (Figures 4 and 5), which may be due to the overproduction of mitochondrial O_2_^−^ in these cellular backgrounds. Typically, cytoplasmic p53 translocation into mitochondria interacts with MnSOD to control the mitochondrial ROS level.^71^ Therefore, the p53 function suppressed by MCT-1 could lead to MnSOD induction and ROS promotion in carcinogenic cells (Figures 4a and b). Upregulation of MnSOD sustains the Warburg effect via mitochondrial ROS generation and AMP-activated kinase activation, which increases the metabolic shift to glycolysis and maintains tumor aggressiveness.^15^ Targeted suppression of MCT-1, YY1 and EGFR capably effectively suppress MnSOD expression in cancer cells (Figures 4f,5b), emphasizing that the amplification of MnSOD signaling via the MCT-1-YY1-EGFR network may determine cancer cell proliferation, invasion and metastasis. Thus, scavenging mitochondrial superoxide formation by modulating the MCT-1-YY1-EGFR-MnSOD axis together with a ROS inhibitor that reduces intracellular and extracellular oxidative stresses might alleviate carcinoma metabolism and metabolic reprogramming of the microenvironment in aggressive tumor.

## Materials and methods

### Cell lines

MCF-10A cells were cultured in DMEM/F-12 complete medium.^27^ A549, H1299 and MDA-MB231 cells were cultured in RPMI 1640 medium.^29^ V5-tagged MCT-1/pLXSN, pCMV p53 and the empty vehicles (pLXSN and pCMV) were transfected into cells as previously described.^29^

The YY1 gene was cloned from A549 cells using a PCR cloning strategy (forward primer: 5′-ATGGCCTCGGGCGACACCCTCTACATCGCCAC-3′ and reverse primer: 5′-CTGGTTGTTTTTGGCCTTAGCATGTGTTAAGA-3′) and inserted into a pLHCX vector with a 3 × FLAG peptide (DYKDDDDK) at the carboxyl terminus. Transfectants carrying pLHCX empty vehicle or FLAG-tagged YY1 vector were maintained in medium containing 50 μg/ml hygromycin.

The pBABE plasmid carrying wild-type EGFR or a mutant EGFR (L858R) gene (Addgene, Cambridge, MA, USA) were transfected into MCF-10A cells, and cells were cultured in 0.5 μg/ml puromycin-containing medium. The H1299 cell variants with wild-type EGFR and EGFR-activating mutants (L858R and exon19 deletion (DEL)) were established as previously described.^72^

MCT-1 expression was suppressed by SureSilencing pGeneClip MCT-1 shRNA (SABiosciences, Valencia, CA, USA) as previously described.^30^ The cells were further transfected with scrambled shRNA or pMKO.1 puro p53 shRNA 2 (Addgene) to deplete p53. Similarly, YY1 and EGFR were, respectively, inhibited in cells using SureSilencing YY1 shRNA (SABiosciences) and EGFR shRNA (SABiosciences) with a TransIT-LT1 transfection reagent (Mirus Bio LLC, Madison, WI, USA) according to the manufacturer’s instructions.

### Antibodies (Abs)

Abs recognizing p53 (DO-1; Santa Cruz Biotechnology, Santa Cruz, CA), GAPDH, β-actin (Abcam, Cambridge, MA, USA), CD31 (BD Pharmingen, San Diego, CA, USA), MnSOD (Enzo Life Sciences, Inc., Farmingdale, NY, USA), p47^phox^ (Signalway Antibody, Baltimore, MD, USA) and integrin beta-1 (CD29; BD Transduction Laboratories, Lexington, KY, USA) were purchased. Abs against phospho-p53 (Ser15), phospho-EGFR (Tyr1068), EGFR and H2AX (Ser139) were obtained from Cell Signaling Technology (Danvers, MA, USA). The Abs against V5-epitope (Invitrogen, Waltham, MA, USA) and intrinsic MCT-1 (N1C3) GeneTex (Irvine, CA, USA) were purchased. Western blot analysis was performed as previously described.^30^

### Apoptosis analysis

A FITC-Annexin V Apoptosis Detection Kit (BD Biosciences, San Jose, CA, USA) was used to analyze apoptosis. The cells were incubated in complete medium with 100 μm hydrogen peroxide (H_2_O_2_) for 24 h or pre-treated with 5 μm AG1478 for 1 h before H_2_O_2_ exposure as indicated.^73^ All the cells were re-suspended in 1 × binding buffer (1 × 10^6^ cells/ml) and reacted with Annexin V-FITC for 15 min. Propidium iodide was used as a counterstain to discriminate necrotic/dead cells. The results were analyzed using a BD FACSCalibur flow cytometer (Becton-Dickinson, San Jose, CA, USA). Annexin V-FITC binding was detected by flow cytometry (Ex=488 nm; Em=350 nm) using a FITC signal detector (FL1), and propidium iodide staining was detected using a phycoerythrin emission signal detector (FL2). The results were analyzed using BD CellQuest Pro Analysis software (BD Biosciences).

An *In Situ* Cell Death Detection kit (Roche, Mannheim, Germany) was used to evaluate DNA fragmentation in apoptotic cells with terminal deoxynucleotidyl transferase dUTP nick-end labeling (TUNEL) followed by flow cytometry, according to the manufacturer’s instructions.

### Promoter activity assay

A YY1 promoter (−1514 to +54) fragment was cloned from A549 cells and inserted into a pGL3-Luciferase basic vector between Nhe*I* and Xho*I* restriction sites. The EGFR promoter vector was constructed as previously described.^74^ The p53 promoter fragment (−188 to +23) was cloned from MCF-10A cells by PCR amplification using the forward primer 5′-cgagctcgtcggcgagaatcctgact-3′ (−188 to −170) and the reverse primer 5′-ggaagcttGGACGGTGGCTCTAGACTTT-3′ (+3 to +23) and then cloned into a pGL3-Luciferase vector between Sac*I* and Hind*III* restriction sites. Luciferase reporter activity was measure as previously described.^27^

### Examination of intracellular ROS and mitochondrial superoxide

DCFH-DA (2′,7′-dichlorofluorescein diacetate; Sigma-Aldrich, St. Louis, MO) and MitoSOX Red (Molecular Probes, Eugene, OR, USA) were used to measure cellular ROS and mitochondrial superoxide, respectively. Cells were incubated with 50 μm DCFH-DA or 5 μm MitoSOX at 37 °C for 30 min. The emitted DCF and MitoSOX fluorescence were quantified using a FACSCalibur flow cytometer (Becton-Dickinson) with excitation/emission wavelengths of 485/530 nm to measure DCF in the FL1 channel and with excitation/emission wavelengths of 510/580 nm to measure oxidized MitoSOX Red in the FL2 channel.

### Tumor progression and immunohistochemistry study

The A549 cells carrying the pcDNA3.1/luciferase vector (1 × 10^6^/100 μl PBS) were inoculated subcutaneously (s.c.) into 6-week-old female BALB/c nude mice (BALB/cAnN.Cg-Foxn1^nu^/CrlNarl; *n*=11), and tumors were allowed to develop for 8 weeks. A randomized method was used to assign the mice into the experimental groups (control and MCT-1). The animal studies were conducted in accordance with the Animal Use Protocol approved by the National Health Research Institutes (NHRI-IACUC-104020-A). Tumor tissues were processed for immunohistochemistry analysis as previously described.^27^ Luciferin (150 mg/kg; PerkinElmer, Waltham, MA, USA) was intraperitoneally injected into mice to detect tumor progression using a Xenogen IVIS 200 bioluminescence imaging system (Caliper LifeSciences, Hopkinton, MA, USA).

The Abs for immunohistochemistry were diluted as follows: MCT-1, 1:500 (GeneTex, GTX117793); YY1, 1:400 (GeneTex, GTX62783); CD31, 1:200 (Abcam, ab28364); CD163, 1:100 (Abcam, ab189915); αSMA, 1:4000 (GeneTex, GTX112862); MnSOD, 1:2000 (Enzo Life Sciences, Inc., ADI-SOD-111-D); p53, 1:100 (Millipore, DAM1698716, Billerica, MA, USA); and phospho-EGFR, 1:100 (Cell Signaling Technology, #4407S).

### Quantitative real-time PCR

Real-time PCR was conducted using SYBR Green Master Mix and analyzed using a LightCycler PCR detection system (ABI PRISM-7900) as described previously.^29^ The primers for MCT-1 (forward: 5′-AGGCATTATCTTCATGCTGTCA-3′; reverse: 5′-AATGATGGGCTGTGGCATAT-3′), YY1 (forward: 5′-GGAACAAGGGCTCTCA AACC-3′ reverse: 5′-CCCGGCAAGTGTGAGTG-3′), EGFR (forward: 5′-CTCCGTTTCTTCTTTGC CCAG-3′), p53 (forward: 5′-TTCCTCTTCCTACAGTACTCC-3′; reverse: 5′-GACGCGGGTGC CGGGCGG-3′), MnSOD (forward: 5′-AGCTATTTGGAATGTAATCAACTGG-3′ reverse: 5′-TAAGCAACATCAAGAAATGCTACA-3′), CD31 (forward: 5′-TAATACAACATCCACGAGGGTC-3′; reverse: 5′-CTGACAGTGTCTTGAGTGGG-3′), CD163 (forward: 5′-CCGGGAGATGAATTCTTGCCT-3′; reverse: 5′-AGACACAGAAATTAGTTCAGCAGCA-3′), α-SMA (forward: 5′-GCTAGAGACAGAGAGGAGCAGG-3′ reverse: 5′-CTCTCTGTCCACCTTCCAGC-3′) and β-actin (forward: 5′-CACCAGGGCGTGATG GTGGG-3′ reverse: 5′-GATGCCTCTCTTGCTCTGGGC-3′) were designed according to the NCBI Probe database.

The half-life of YY1 mRNA was measured when the A549 cells were treated with 5 μg/ml actinomycin D (Sigma-Aldrich) for various times, and the remaining YY1 mRNA was analyzed by quantitative real-time PCR.

### Subcellular fractionation

A Mitochondria/Cytosol Fractionation Kit (Abcam, Cambridge, MA, USA) was used to perform subcellular fraction. Cells (5 × 10^6^ cells) were incubated in 1 × Cytosol Extraction Buffer Mix containing DTT and protease inhibitors, homogenized in an ice-cold Dounce tissue grinder and centrifuged at 700 *g* in a microcentrifuge for 10 min at 4 °C. The nuclear pellet was kept, and the supernatant was further centrifuged at 10000 *g* for 30 min at 4 °C. The supernatant (cytosolic fraction) was collected, and the pellet (mitochondria) was re-suspend with Mitochondrial Extraction Buffer to isolate mitochondrial proteins. Nuclear proteins were extracted with 1 × SDS-PAGE sample buffer.

A Subcellular Protein Fractionation kit (Thermo Fisher Scientific Inc., Waltham, MA, USA) was used to characterize cytosolic and membrane proteins. Cells (5 × 10^6^ cells) were incubated in cytoplasmic extraction buffer on ice for 10 min. The supernatant was centrifuged at 500 *g* for 5 min to collect the cytosolic fraction. The pellet was incubated in membrane extraction buffer on ice for 10 min and centrifuged at 3000 *g* for 5 min to collect the membrane fraction.

### Cell invasion assay

Cell invasiveness was examined using Corning BioCoat Tumor Cell Invasion Systems (Corning, Corning, NY, USA). The cells (5 × 10^4^) were incubated with serum-free medium and 6 μm DPI (Sigma-Aldrich) or DMSO in the top insert and with 10% FBS-containing medium in the bottom chamber for 24 h. Cells invading to the lower chamber were fixed with methanol, stained with crystal violet and counted using a microscope and the × 20 objective lens.

### Gene expression levels in TissueScan Lung Cancer tissue array

TissueScan Lung Cancer Tissue qPCR Panels (II, III and V; OriGene Technologies, Inc., Rockville, MD, USA) were used to analyze MCT-1, YY1, EGFR, MnSOD, p53, CD31, CD163 and α-SMA mRNA levels by quantitative RT-PCR analysis. The relative mRNA levels were calculated using the formula: ΔC_T_ =C_t_ normal tissue group–C_t_ cancer group. The fold change in each gene was calculated using the formula 2^−^^ΔCT^. Clinical studies were approved by the Institutional Review Board (IRB) of the National Health Research Institutes (EC1031216-W).

### Statistics

Student’s *t*-test was used to compare the mean of the control group and the experimental groups. *X*^2^ tests were used to assess differences between cancer stages and different cancer types and normal tissues. The Phi coefficient was used to evaluate the relationship between high expression of MCT-1 and YY1. Pearson’s coefficient correlation was used to evaluate the relationship between MCT-1 and YY1, EGFR, MnSOD, p53, CD31, CD163 and α-SMA. Correlations between MCT-1 expression levels and overall survival, recurrence-free survival and EGFR mutations in the Okayama data set were analyzed using Student’s *t*-test. A *P*-value <0.05 was considered statistically significant.
