# Supplementary material for: Oncogenic MCT-1 activation promotes YY1-EGFR-MnSOD signaling and tumor progression
Source: Oncogenesis. 2017 Apr 10;6(4):e313–. doi: 10.1038/oncsis.2017.13 (PMC5520490; doi:10.1038/oncsis.2017.13)

Supplementary FIG. 1. MCT-1 overexpression and p53 knockdown together enhance YY1 and EGFR expression. MCF-10A cells with different MCT-1 and p53 expression levels were analyzed. **(a)** The relative p53 mRNA level was assayed after p53 knockdown. **(b)** The relative EGFR promoter activity was compared between different p53 backgrounds. **(c)** The relative EGFR mRNA level was examined in different p53 contents. **(d)** YY1 and EGFR proteins were characterized in cells that had normal p53 levels (control, MCT-1) or reduced p53 levels (control/-p53, MCT-1/-p53). The YY1, EGFR and p53 protein levels were normalized to GAPDH and compared with scrambled knockdown (lane 1). The data represent the mean±SD (n=3). **$p<0.01$; ***$p<0.001$.

Supplementary Fig. 1

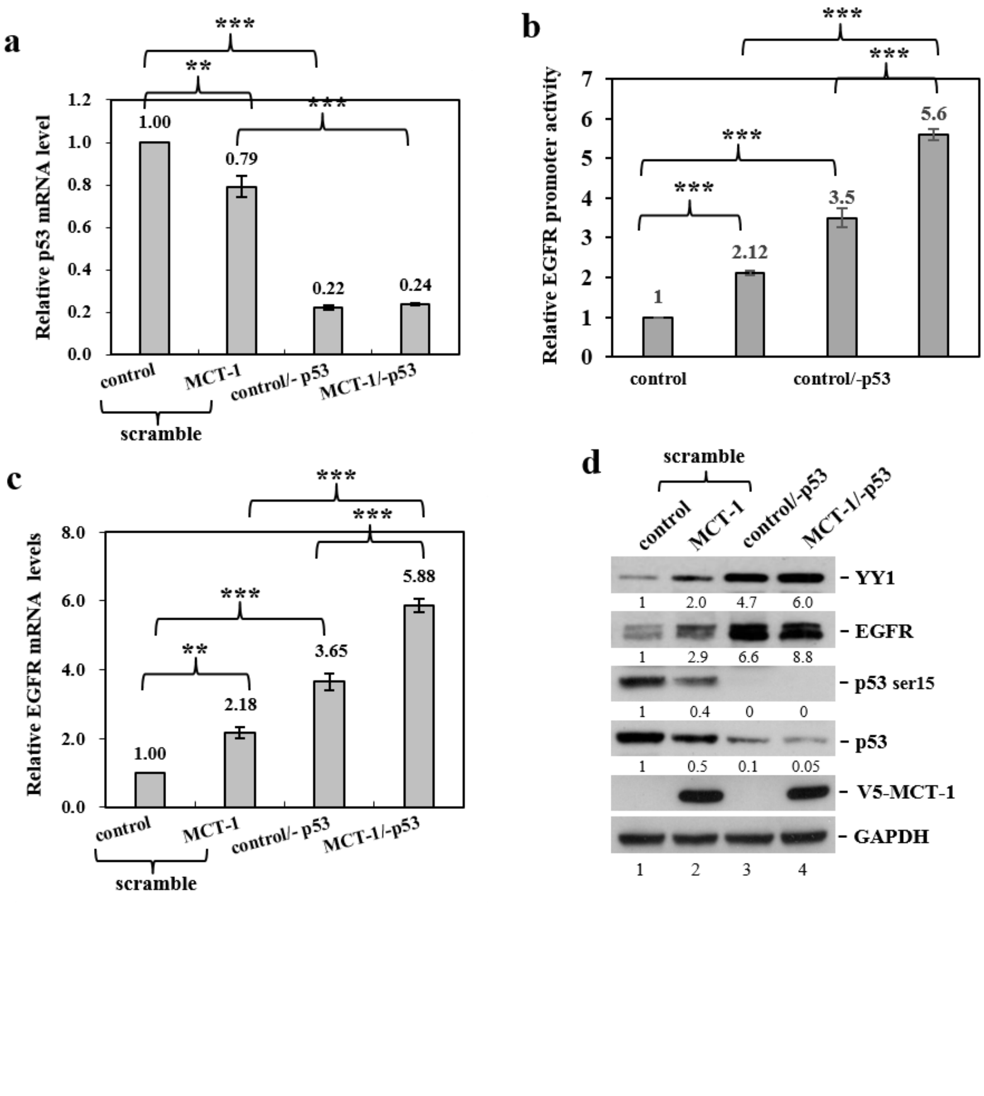

Supplementary FIG. 2. Knockdown of YY1 induces apoptosis. (a) Apoptotic events were examined by TUNEL assays in A549 cells with different MCT-1 and YY1 levels. Data represent the mean±SD (n=3). *p<0.05; **p<0.01; ***p<0.001. **(b)** The protein expression levels were assayed after H_2O_2 exposure for 1 h or AG1478 co-treatment in MCF-10A cells. **(c)** The expression and phosphorylation levels of EGFR were examined before or after introducing wild-type EGFR. **(d)** The MCT-1 promoter region contains several putative binding sites of YY1. **(e)** EGFR, MnSOD and MCT-1 expression levels were assayed in A549 cells with YY1 silencing (shYY1) or scrambled knockdown.

Supplementary Fig. 2

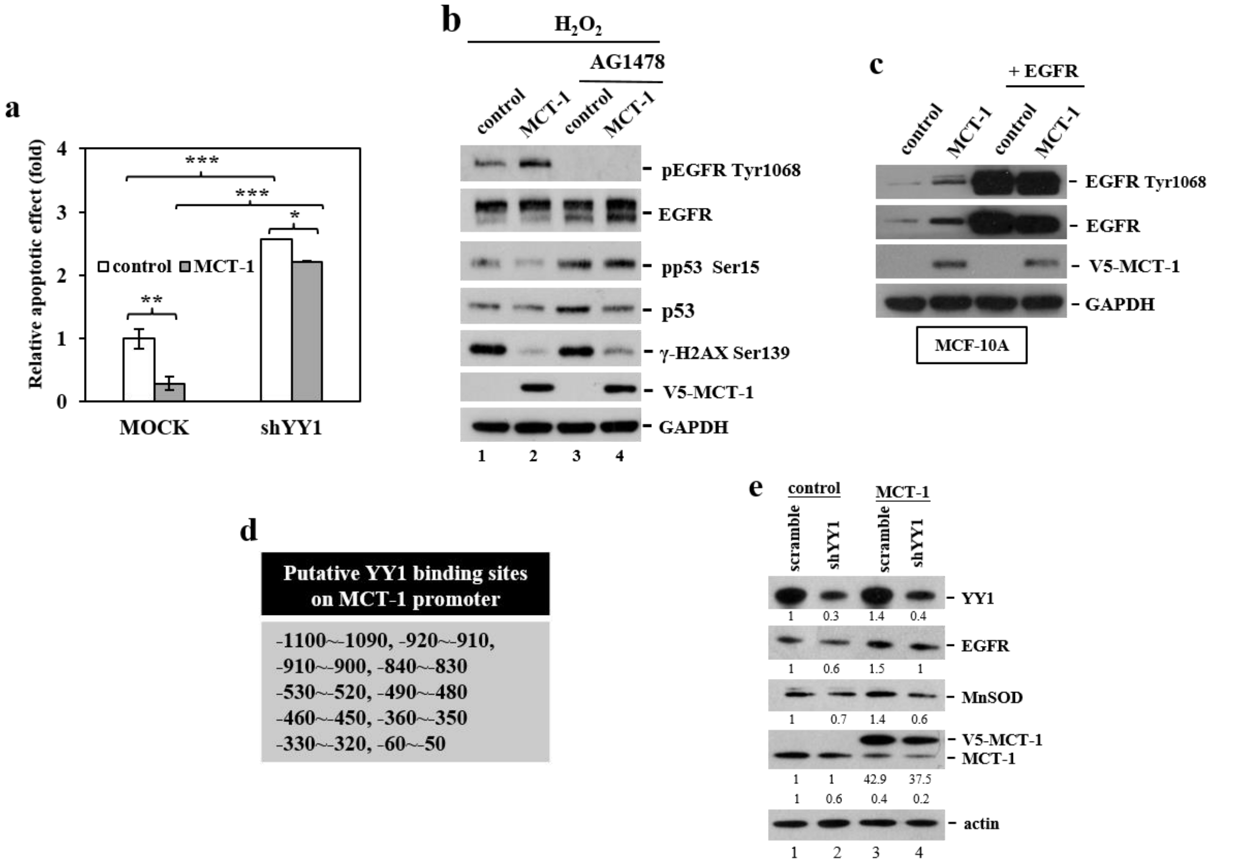

Supplementary FIG. 3. Relative MCT-1 mRNA expression levels in human lung cancer.

Using the ONCOMINE database (www.oncomine.org), we found that the MCT-1 gene was highly activated in different subtypes of lung cancer in the Hou dataset and in lung adenocarcinomas of the Okayama and Wei datasets compared with normal lung tissues.

Supplementary Fig. 3

Relative MCT-1 expression level in human lung cancers

Dataset and cancer type [1]	Total	MCT-1 expression fold (p-value)
**Hou dataset**	156	
Adenocarcinoma	45	1.880 (7.08E-14)
Squamous	27	1.959 (1.00E-10)
Large cell	19	1.615 (4.16E-5)
**Okayama dataset**	246	
Adenocarcinoma	226	1.98 (2.29E-19)
**Wei dataset**	50	
Adenocarcinoma	45	2.268 (1.10E-11)

[1] Using the datasets of Oncomine database (www.oncomine.org).

Supplementary FIG. 4. Clinical relevance of YY1, EGFR, MnSOD and p53 expression in human lung cancer. TissueScan lung cancer tissue cDNA arrays were analyzed for the mRNA levels of YY1 **(a)**, EGFR **(b)**, MnSOD **(c)** and p53 **(d)**. The mRNA level identified in each sample was normalized to the β-actin mRNA level and calibrated with the average mRNA level in normal lung tissue. The mRNA levels with a 1.5-fold increase over normal lung tissue were defined as "high" expression of the gene. The mRNA levels with a 1.5-fold decrease compared with the normal tissue were considered "low" expression of the gene. (**e)** The dot plot shows the $\log_2$ ratio of the indicated gene in lung tumors (T) vs. normal lung tissues (N) after normalizing to the β-actin mRNA level. Chi-square tests evaluate the clinical relevance of the indicated gene expression at different lung cancer stages relative to normal lung tissue.

Supplementary Fig. 4

a

YY1 mRNA level in lung cancer

Stage	$YY1^{high}$	$YY1^{low}$	p-value
normal	0	13 (100%)	-
I	32 (67%)	16 (42%)	<0.0001
II	11 (37%)	19 (77%)	<0.01
III/IV	19 (41%)	27 (63%)	<0.01
Total	62 (50%)	62 (50%)	<0.01

b

EGFR mRNA level in lung cancer

Stage	$EGFR^{high}$	$EGFR^{low}$	p-value
normal	0	13 (100%)	-
I	25 (69%)	11 (31%)	<0.0001
II	19 (86%)	3 (14%)	<0.0001
III/IV	19 (59%)	13 (41%)	<0.0001
Total	63 (70%)	27 (30%)	<0.0001

c

MnSOD mRNA level in lung cancer

Stage	$MnSOD^{high}$	$MnSOD^{low}$	p-value
normal	0	13 (100%)	-
I	28 (68%)	13 (32%)	<0.0001
II	21 (81%)	5 (19%)	<0.0001
III/IV	26 (67%)	13 (33%)	<0.0001
Total	75 (71%)	31 (29%)	<0.0001

d

p53 mRNA level in lung cancer

Stage	$p53^{high}$	$p53^{low}$	p-value
normal	13 (100%)	0	-
I	4 (8%)	44 (92%)	<0.0001
II	5 (17%)	25 (83%)	<0.0001
III/IV	1 (2%)	45 (98%)	<0.0001
Total	10 (8%)	114 (92%)	<0.0001

e

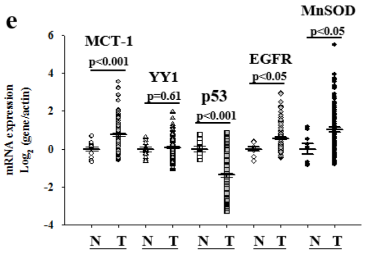

Supplement: Supplementary Figure Legends and Figures [file oncsis201713x1.pdf]
